# The importance of the surface roughness and running band area on the bottom of a stone for the curling phenomenon

**DOI:** 10.1038/s41598-020-76660-8

**Published:** 2020-11-26

**Authors:** Takao Kameda, Daiki Shikano, Yasuhiro Harada, Satoshi Yanagi, Kimiteru Sado

**Affiliations:** 1 Snow and Ice Research Laboratory, Kitami Institute of Technology, 165 Koencho, Kitami, Hokkaido 090-8507 Japan; 2 Optical Engineering Laboratory, Kitami Institute of Technology, 165 Koencho, Kitami, Hokkaido 090-8507 Japan; 3 Hokkaido Kushiro Meiki Senior High School, 1-38-7 Aikoku-nishi, Kushiro, Hokkaido 085-0057 Japan; 4 Professor Emeritus, Kitami Institute of Technology, 165 Koencho, Kitami, Hokkaido 090-8507 Japan; 5 Present Address: Utsunomiya Management Office, East Nippon Expressway Co., Ltd, 24-2 Moro, Kanuma, Tochigi, 322-0026, Japan

**Keywords:** Physics, Applied physics

## Abstract

Curling is a sport in which players deliver a cylindrical granite stone on an ice sheet in a curling hall toward a circular target located 28.35 m away. The stone gradually moves laterally, or curls, as it slides on ice. Although several papers have been published to propose a mechanism of the curling phenomenon for the last 100 years, no established theory exists on the subject, because detailed measurements on a pebbled ice surface and a curling stone sliding on ice and detailed theoretical model calculations have yet to be available. Here we show using our precise experimental data that the curl distance is primarily determined by the surface roughness and the surface area of the running band on the bottom of a stone and that the ice surface condition has smaller effects on the curl distance. We also propose a possible mechanism affecting the curling phenomena of a curing stone based on our results. We expect that our findings will form the basis of future curling theories and model calculations regarding the curling phenomenon of curling stones. Using the relation between the curl distance and the surface roughness of the running band in this study, the curl distance of a stone sliding on ice in every curling hall can be adjusted to an appropriate value by changing the surface roughness of the running band on the bottom of a stone.

## Introduction

Curling is a sport in which players deliver a cylindrical granite stone (about 28 cm in diameter and 18 to 19 kg in weight) on an ice sheet in a curling hall toward a circular target located 28.35 m away. The stone gradually moves laterally, or curls, as it slides on ice. The exact origin of curling is unknown, but an old curling stone engraved with the date 1511 was discovered in Scotland^1,2^, and two oil paintings dated 1565 by a sixteenth century Flemish painter, Pieter Bruegel the Elder (c. 1525–1569), portrayed an activity similar to curling being played on frozen ponds^2,3^. The first written evidence of curling appeared in 1540 when a notary in Scotland recorded in his protocol book a challenge between two persons who threw a stone on ice^3^. Curling in its early days was played on frozen lochs and ponds during a harsh European winter. Curling has now evolved into a popular modern sport that takes place on indoor ice rinks with the condition and temperature of the ice carefully controlled.

A curling player releases a stone with a small degree of clockwise rotation on ice, and the stone gradually curls toward the right, while a small anticlockwise rotation allows it to curl toward the left. When a player releases a stone without any rotation, the stone usually starts to rotate by itself and the trajectories are different every time. The curling phenomenon is primarily important in curling games because the amount of lateral displacement, called the curl distance, contributes to the strategy of games. Since the first scientific paper by Harrington^4^, several papers^5–27^ have been published to propose a mechanism of the curling phenomenon. However, no established theory exists on the subject, because detailed measurements on a pebbled ice surface and a curling stone sliding on ice and detailed theoretical model calculations have yet to be available.

The surface conditions of ice and a stone in curling games have distinctive features. The ice surface is not flat but has small ice pebbles attached to a flat ice surface by spraying water droplets onto the ice. The tops of the pebbles are cut off with a blade called a nipper. The bottom of a curling stone is concave at its centre, with only a running band, an annulus with an inner diameter of about 125 mm and a width of about 3−7 mm, touching the ice. These features reduce the contact area between the ice surface and the stone.

The curling behaviour of a stone sliding on ice has been explained by three models: a left–right asymmetry model (LR model, with different frictional forces on the left and right sides of a stone)^4–7^, a front-back asymmetry model (FB model, with different frictional forces on the front and back sides of a stone)^8–24^ and a pivot-slide model (PS model, where brief pivots of a stone around a point between a stone and pebbles cause the curling behaviour)^25–27^.

Because the LR models failed to explain why a curling stone curls^8^, the FB and the PS models have been developed. The FB models are divided into six models, in which different mechanisms have been proposed for different front and back frictional forces: a pressure difference model (a larger pressure works on the front running band than on the back running band)^9–11^, a water layer model (a water layer is assumed to be produced by frictional heating, and the layer reduces the frictional forces at the front running band)^12–14^, a snowplow model (small ice debris are formed by a stone and accumulate at the front running band)^15^, an evaporation-abrasion model (with an asymmetrical effect on evaporation and abrasions on the tops of pebbles and the effects of ice debris)^16–18^, a scratch-guiding model (guiding of small scratches left on the tops of pebbles by a stone)^19–22^ and an edge model (with different rake angles of a stone at the front and back of the stone)^23,24^. Recent papers discuss a possibility of the PS model^25–27^, which was originally suggested by Penner^8^. Scientific discussions between different modellers have been raised and published^28−35^. However, no established theory exists, because detailed measurements on a pebbled ice surface and a curling stone sliding on ice and detailed theoretical model calculations have yet to be available, as partially explained in review papers^2,36,37^.

The curl distance for a stone with a typical angular velocity (four turns during 28.35 m of movements for 23 s; this is equivalent to 1.09 rad/s = 62.6°/s) ranges from about 0.5 to 1.5 m at a circular target located 28.35 m away on ice. The differences in ice surface conditions (ice surface temperature, the number density of pebbles, and the diameter and height conditions of pebbles) and curling stones are the factors for controlling the curl distances. Thus, to determine the factors affecting the curling distances by precisely measuring these conditions, we carried out our measurements at ADVICS Tokoro curling hall in Kitami, Japan.

## Methods

The room temperature at a height of 1.5 m in the ADVICS Tokoro curling hall was 5 to 7 °C, and the ice surface temperature was − 3.5 to − 2.5 °C during our experiments.

We used four instruments in this study: an automatic tracking total station (ATTS, Type S7, Nikon-Trimble Co. Ltd., Japan), a probe-type surface roughness meter (SJ-210, Mitutoyo Corporation, Japan), a small digital camera (TG-4, Olympus Corporation, Japan), and a confocal laser scanning microscope (VK-9700, Keyence Corporation, Japan).

**Figure 1:**
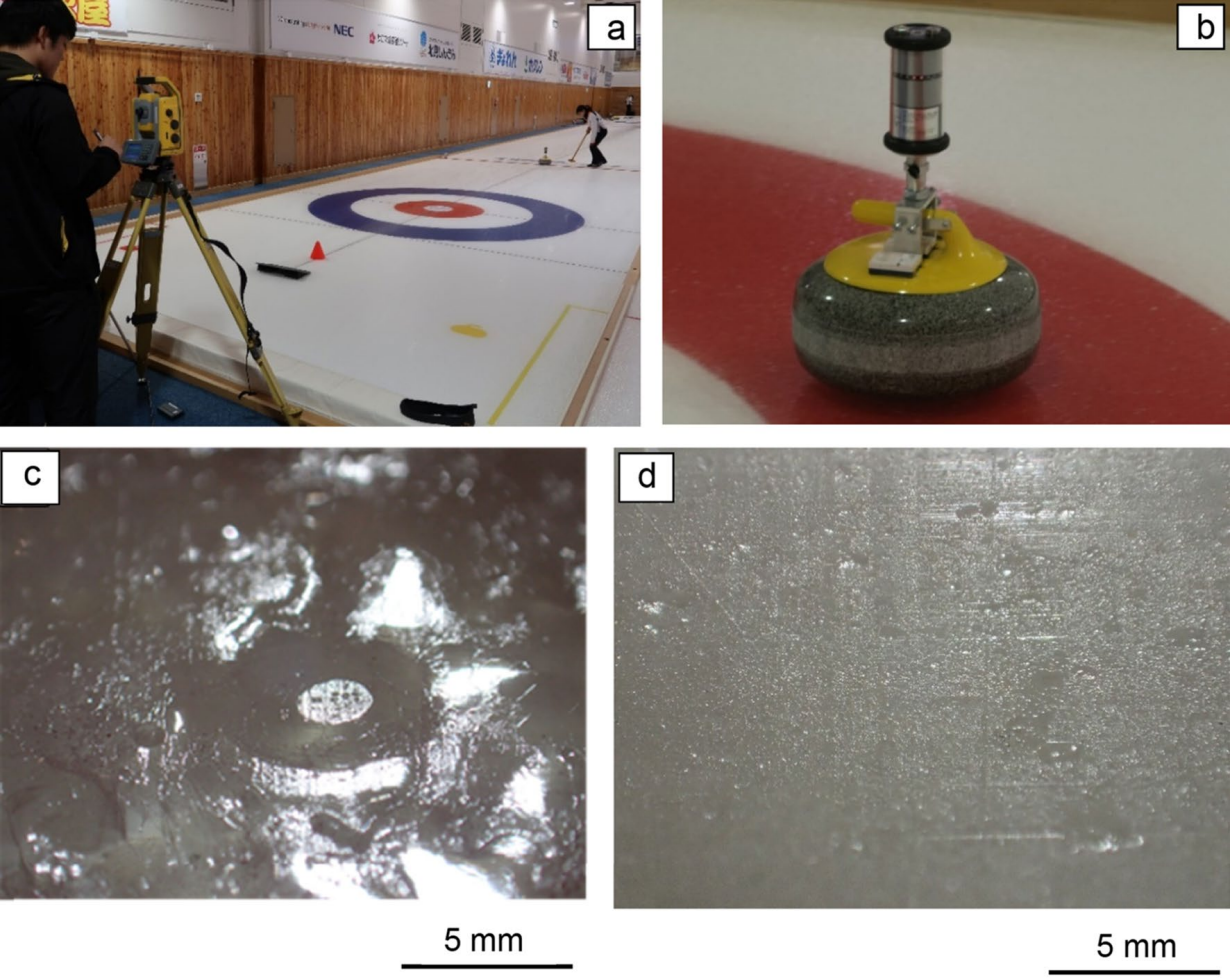
(**a**) Auto-tracking total station (ATTS) for measuring the stone’s position moving on ice; (**b**) A cylindrical target for the ATTS fixed on the centre of a stone using a special device; (**c**) Ice surface condition of a pebbled ice surface just after the nipper operations; and (**d**) A flat ice surface without pebbles specially designed for our experiments.

The ATTS was used for measuring the trajectory of the centre of a curling stone sliding on ice as shown in Fig. 1a. The accuracy of the position in a static condition was  ±2 mm and the average time interval for the positional data of the stone was 0.4 s. An active prism (Model T-360SL LED target, Nikon-Trimble Co. Ltd., Japan, 55 mm in diameter, 135 mm in height and 520 g) was placed on the centre of the stone with a special device shown in Fig. 1b. The prism has a cylindrical shape, instead of a usual polyhedron shape. With the cylindrical prism, we can continuously measure the positions of the stone while the stone is sliding and slowly rotating on ice.

The probe type surface roughness meter was used for measuring the textured condition of the running band of curling stones. The resolution of the roughness height (*z-*direction) is 0.02 μm. The arithmetical mean of the surface roughness (*R*_a_), defined by Eq. (1), was used to express the textured condition:

1 $$R_{{\text{a}}} = \frac{1}{n}\mathop \sum \limits_{i = 1}^{n} \left| {\Delta {\text{z}}_{{\text{i}}} } \right|$$

where *n* is the total number of data points in the measuring direction with a resolution of 1.5 μm, and Δ*z*_i_ is a vertical height variation from the average.

The small digital camera was used for imaging the ice surface of the curling rink. This camera has a microscopic mode with focus stacking for taking clear microscopic photographs of the ice surfaces of curling rinks.

The laser microscope was used for measuring the shapes of pebbles. Replicated samples of pebbles were formed by UV curing resin (type NOA81, Norland Products Inc., USA) with ultraviolet light on the pebbled ice surface in the rink and used for the microscope. The accuracy of the height measurements was  ±0.8 μm. Because the replica samples shrink at 1.1% in the parallel direction of the specimens and 2.7% in the vertical direction of the specimens during solidification^38^, we corrected them for the measurements of the replicated samples. The lower linear shrinkage ratio in the parallel direction was caused by friction between the resin and the specimens, which occurs only in the parallel direction, not in the vertical direction^38^. This method was originally reported in snow crystal studies^38,39^ and applied to this study.

We used four stones (A to D) with different surface roughness conditions. The surface roughness of the running band of a stone was adjusted using a sheet of sandpaper as follows. A sheet of sandpaper (23 by 28 cm) was secured on a flat desk with a double sided tape, and each stone was moved on the paper forth and back in a straight line by a distance of about 10 cm. This procedure was carried out four times each with a stone rotation angle of 45°. When the stone was rotated by 45°, the stone was lifted. A total of four forth-and-back movements were carried out with the same rotation angle on the same sandpaper.

We use a sheet of sandpaper of P80 grit number as a reference. We selected rougher sandpaper (P40) for A2 to increase the surface roughness of A2. We selected smoother sandpaper (P120) for C1 to slightly adjust the surface roughness of C1. For B2 and D1, we use the original surface roughness of the running band, and did not use the above sandpaper operations. Thus, sandpapers with the following grit numbers were used for the respective running bands: P80 for A1, B1, C2, and D2; P40 for A2; and P120 for C1.

Because each stone has two running bands on its top and bottom, we measured the eight running bands of the four curling stones. We used two types of ice surface in our experiments in ADVICS Tokoro curling hall: a typical ice surface with pebbles (Fig. 1c) and a flattened ice surface (Fig. 1d). The typical ice surface was prepared by spraying water droplets onto the ice. The flat ice surface was prepared by cutting pebbles with a special cutting machine Ice King (https://iceking.ca/web/en/). In this paper, we used stone trajectory data measured on August 17 and 18 in 2019, and a total of 201 trajectory data sets were recorded on the days. All stones were released by skilled members of the curling club at Kitami Institute of Technology.

**Figure 2:**
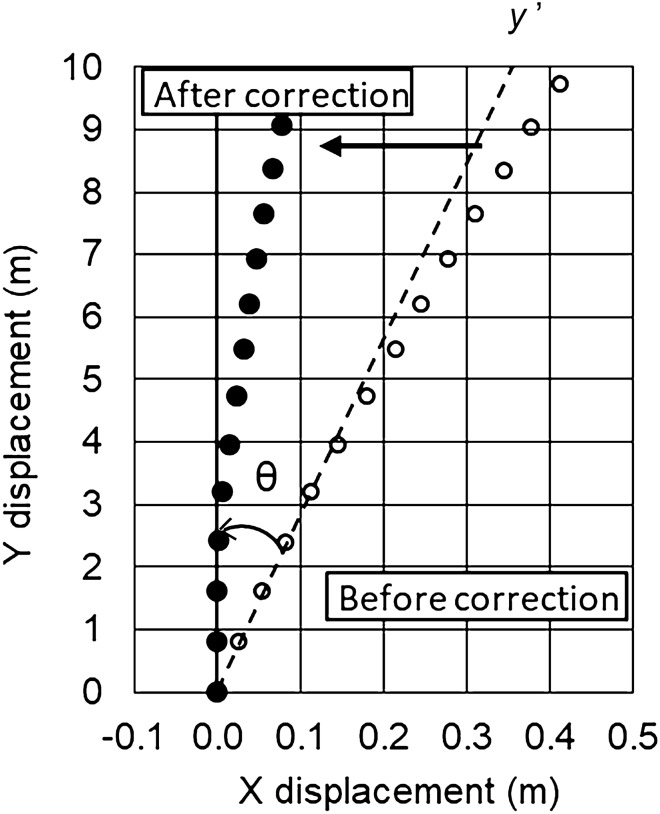
A method for correcting a stone’s trajectories in the delivery direction of *y*ʹ-axis to those in the direction of *y-*axis. The direction of *y*-axis is parallel to the longitudinal direction of the ice rink. Open circles are the original positions (*x*_i_ʹ, *y*_i_ʹ) of the centre of a stone before correction, and solid circles are the positions (*x*_i_, *y*_i_) of the centre of the stone after correction as described in the text.

For the positional data of a curling stone measured by the ATTS, we defined that *y*-axis is parallel to the longitudinal direction of the ice rink, and *x*-axis is vertical to the *y*-axis. A curling stone was normally delivered along the *y*-axis in our experiments; however, it was difficult to perfectly deliver the stone along the *y*-axis. Thus, we corrected the stone’s trajectory data as follows, outlined in Fig. 2.

1. A least square method by a linear approximation was applied to the first 3 to 8 positional data sets of the stone after its delivery and the linear lines were obtained, because a stone moves almost lineally for a few seconds after its delivery.
2. The maximum distances for each data set were calculated from the lines.
3. The most appropriate linear line was selected with the following two criteria: the maximum distance between the lines and the positional data is less than 2 mm (measurement accuracy of the ATTS), and the linear line uses the maximum number of positional data points.
4. The broken line in Fig. 2 is the linear line selected with the above two criteria and expressed in Eq. (2). The open circles in Fig. 2 indicate the original positional data (*x*_i_*′*, *y*_i_*′*) of a stone, and *y′* is the original direction of a stone. A tilt angle (*θ)* between *y′* and *y* axes expressed in Eq. (3) is obtained as follows:
  2 $${y}^{^{\prime}}=a{x}^{^{\prime}}+b$$
  3 $$\theta = {\mathrm{tan}}^{-1}a$$
  where *x′* denotes the axis normal to *y*′.
5. The *x* and *y* axes in Fig. 2 were calculated by the tilt angle *θ* using Eqs. (4) and (5), which are the rotation matrix equations of the coordinates. The corrected positional data of the stone (*x*_i_, *y*_i_) shown by solid circles in Fig. 2 were obtained as follows:
  4 $$x={ x}^{^{\prime}}\mathrm{cos}\theta +{y}^{^{\prime}}\mathrm{sin}\theta$$
  5 $$y=-{x}^{^{\prime}}\mathrm{sin}\theta +{y}^{^{\prime}}\mathrm{cos}\theta$$
  where *x* and *y* are the lateral and longitudinal displacements, respectively.

We defined two types of curl distance for our experimental data. If a stone stopped within 1 m from the centre of the house, 28.35 m away, the curl distance calculated in Eq. (4) was used. If a stone stopped at more than 1 to 2 m from the centre of the house in the *y*-direction, the trajectories of the stone were pulled back by the distances along the *y*-direction, and the curl distances at 28.35 m were corrected by the *x* position at *y* = 0.

All experiments were carried out in accordance with relevant guidelines, regulations and ethics approval by ADVICS Tokoro curing hall in Kitami, Japan. Permission was individually obtained from two persons who appear in Fig. 1a.

## Results and discussion

Figure 1c shows a typical pebble on the ice in ADVICS Tokoro curling hall. The pebble is in the shape of a spherical segment, which is a cutting sphere with a pair of parallel planes. The upper flat surface was initially formed by a nipper, which is a specially designed device to make uniform heights of pebbles, and the nipper is usually used before curling games.

The diameter of the upper surface ranged from 0.36 to 2.18 mm, and the average diameter was 0.97 ± 0.63 mm for seven samples (the value following the ± sign is a standard deviation). The diameter of the lower base of pebbles ranged from 1.57 to 6.84 mm, and the average diameter was 3.58 ± 1.62 mm. The height of pebbles ranged from 0.11 to 0.16 mm, and the average height was 0.13 ± 0.02 mm. We found that the upper surface was slightly enlarged and the height was slightly shortened after stones passed on the pebbles. The average number density of pebbles ranged from 2 to 5 cm^−2^. Figure 1d shows the flat ice surface in ADVICS Tokoro curling hall. This ice surface was formed by an Ice king, which is usually used for removing the pebbles and levelling the ice surface on curling rinks. The flat ice surface is not used for ordinary curling games and is specially designed for our experiments.

**Figure 3:**
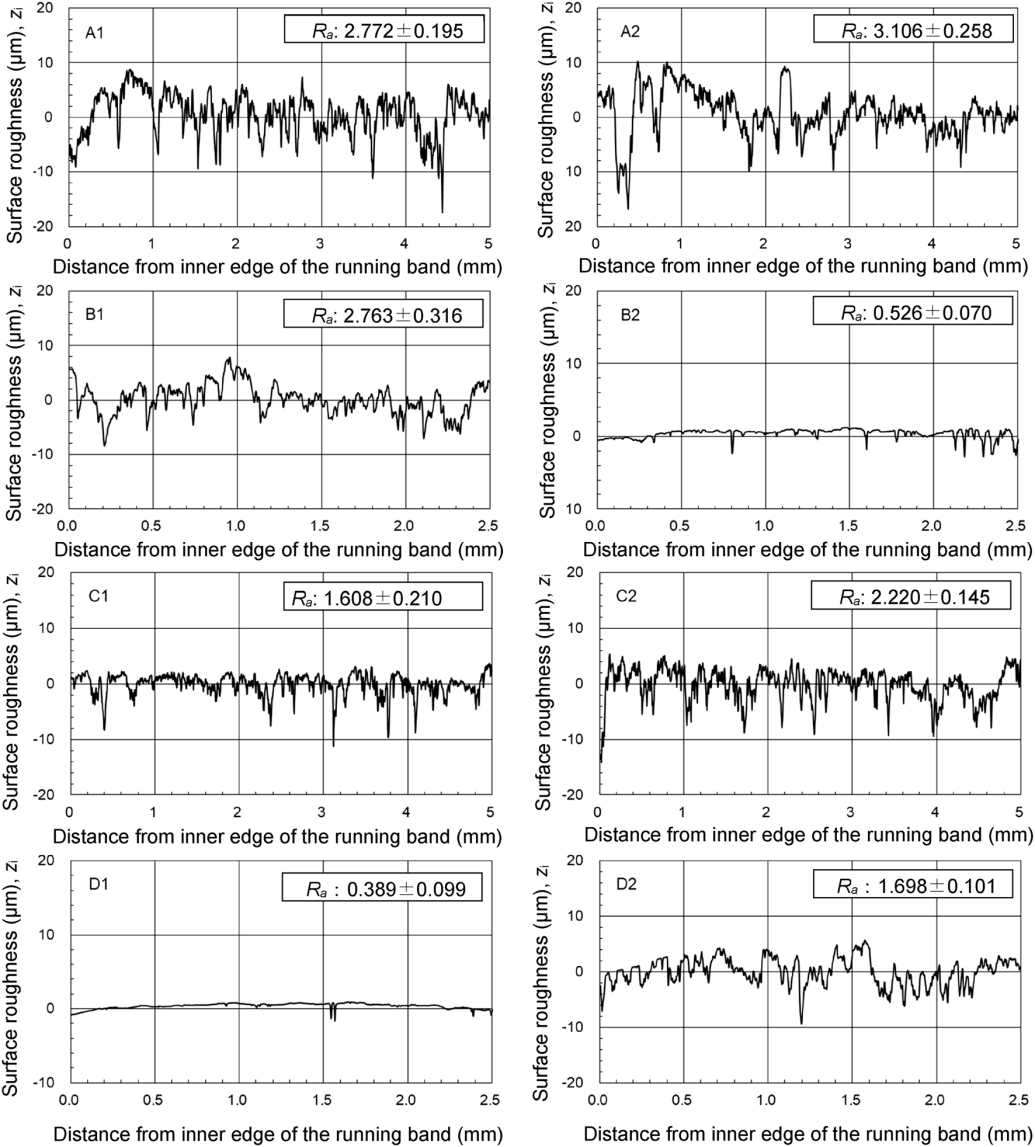
Surface roughness profiles of eight running bands for four stones. Average surface roughness *R*_a_ (average value ± standard deviation) is expressed at the top right corner in each panel.

We measured the surface roughness profiles of each running band at four places at 90° intervals of the four stones using the probe type surface roughness meter. Thus, the total of 32 surface roughness profiles of the running bands (4 stones × 2 surfaces × 4 places) were measured. Figure 3 shows examples of surface roughness profiles for the eight running bands of the four stones. The average value *R*_a_ ranges from 0.389 ± 0.099 μm to 3.106 ± 0.258 μm as shown in Fig. 3. Table 1 summarizes the surface roughness data of four stones.

**Table 1:** Average surface roughness (*R*_a_) of four stones used in this study.

Stone	Running band	Average ± standard deviation (μm)
A	A1	2.772 ± 0.195
	A2	3.106 ± 0.258
B	B1	2.763 ± 0.316
	B2	0.526 ± 0.070
C	C1	1.608 ± 0.210
	C2	2.220 ± 0.145
D	D1	0.389 ± 0.099
	D2	1.698 ± 0.101

**Figure 4:**
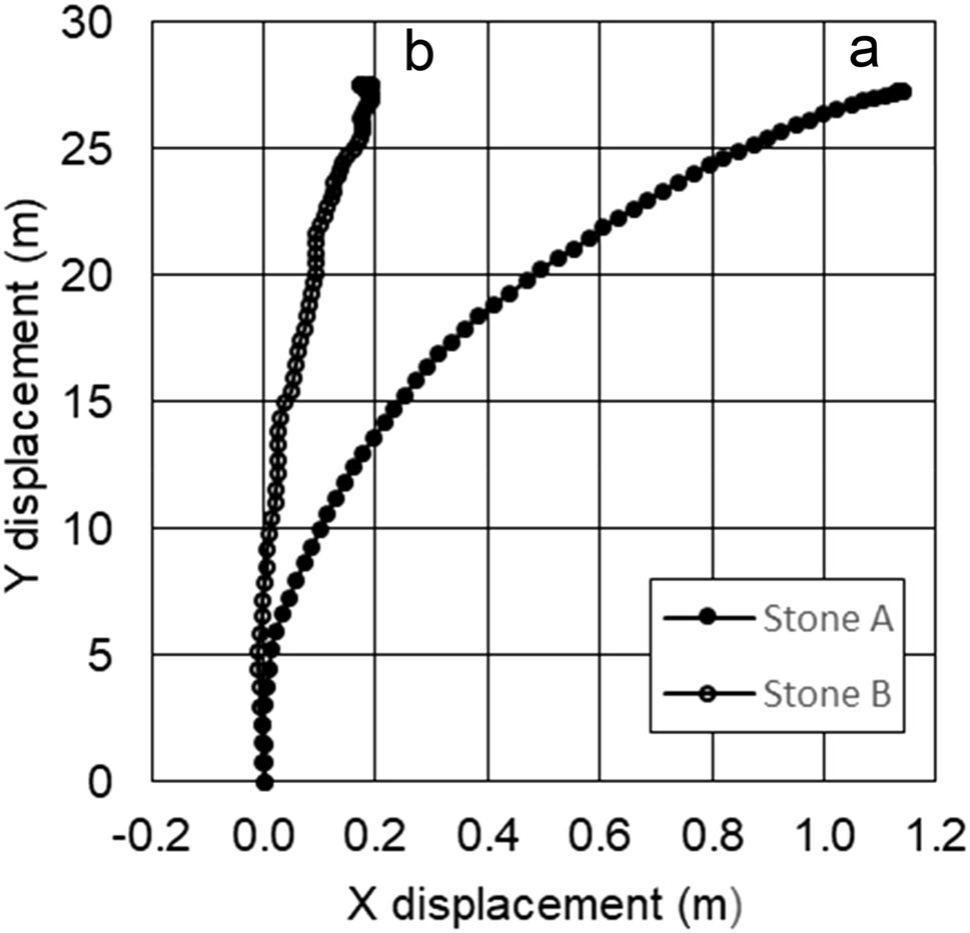
Typical trajectories of stones moving on ice. Surface roughness of stones A1 and B2 are 2.772 ± 0.195 μm and 0.526 ± 0.070 μm, respectively. These trajectories were measured at ADVICS Tokoro curling hall on August 17, 2019.

Figure 4 shows typical examples of the trajectories of stone A with the running band A1 (*R*_a_: 2.772 ± 0.195 μm) and stone B with the running band B2 (*R*_a_: 0.526 ± 0.070 μm), which were obtained after the coordinate rotations described in Eqs. (4) and (5). The two stones were similarly rotated clockwise, by about four turns during the 28.35 m movements, which is equivalent to about 1.07 rad/s = 61.3°/s. Stone A1 is a normal curling stone used in ordinary curling games, and stone B2 is a special stone having a running band with smoother surface roughness.

We found that stone A1 moves along the almost straight trajectory within 2 m after delivery and gradually curls to the right, with the curl distance of 1.1 m at 28.35 m away. On the other hand, the curl distance of stone B2 is 0.2 m, which is 1/5 of that of stone A1, and stone B2 moves with a complex trajectory as shown in Fig. 4b. We measured the trajectories of stone B2 several times, which were found different every time. It seems that the trajectories of stone B2 were affected by accidental collisions of some pebbles on the ice.

**Figure 5:**
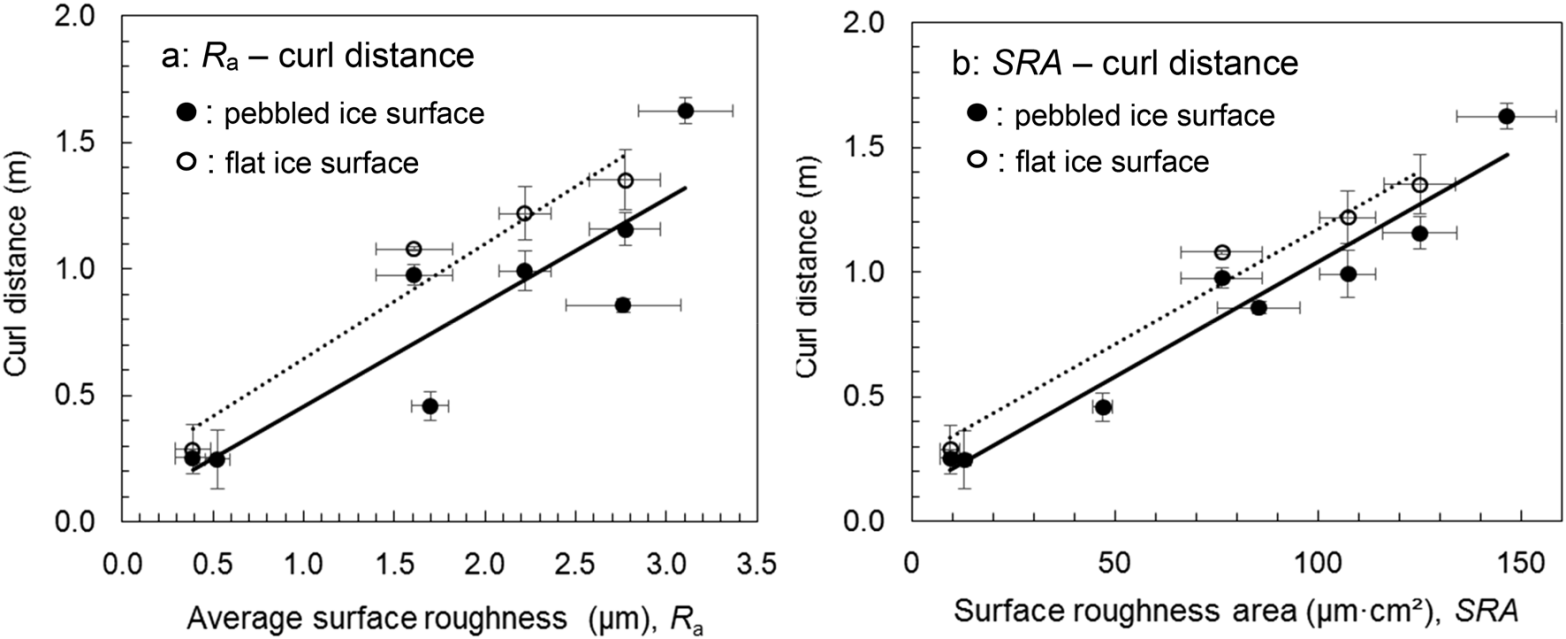
(**a**) Relationship between average surface roughness *R*_a_ of a stone and the curl distances for ordinary pebbled ice surface shown by solid circles and a solid line, and flat ice surface without any pebbles shown by open circles and a dashed line. (**b**) Relationship between surface roughness area (*SRA*) of a stone and the curl distances for ordinary pebbled ice surface shown by solid circles and a solid line, and flat ice surface without any pebbles shown by open circles and a dashed line. Error bars show the ranges of standard deviation.

Figure 5a shows the relationship between the average surface roughness *R*_a_ for different curling stones and the curl distance for an ordinary pebbled ice surface shown by solid circles and a solid line, and for a flat ice surface shown by open circles and a broken line at a typical angular velocity (about three to five turns from its delivery to stop; the initial angular velocity of the stone ranges from 50 to 80°/s). A total of 43 trajectory data sets were used in Fig. 5a,b. We found that the curl distance increases with increasing surface roughness, and the regression lines are given by

6 $${x}_{\mathrm{p}}= 0.411{R}_{\mathrm{a}}+0.046$$

7 $${x}_{\mathrm{f}}= 0.453{R}_{\mathrm{a}}+0.193$$

where *x*_p_ and *x*_f_ denote the curl distances for the normal pebbled ice surface and for the flat ice surface, respectively. The correlation coefficient (*r*) and the level of significance (*p*) are 0.88 (*p* < 0.01) for the normal pebbled ice surface, and 0.97 (*p* < 0.01) for the flat ice surface.

The maximum difference between the curl distances *x*_p_ and *x*_f_ was 0.273 m for *R*_a_ = 3 μm using Eqs. (6) and (7). Because the contact area between the running band and the ice becomes larger for the flat ice surface and the frictional force between the stone and the ice surface will increase, the curling force of a stone seems to be larger.

Because the width of the eight running bands (*d*) is slightly different for each stone, we define the surface roughness area (*SRA*) for each running band using Eq. (8):

8 $$SRA={ R}_{\mathrm{a}} A = {\pi R}_{\mathrm{a}} \left(\overline{r}_{2}^{\ \ 2}-\overline{r}_{1}^{\ \ 2}\right)= {\pi R}_{\mathrm{a}} \overline{d} (2\overline{r}_{1}+\overline{d})$$

where *A* is the surface area of the running band, $\overline{r}_{1}$ and $\overline{r}_{2}$ are the average inner and outer radii of the running band, and $\overline{d}$ is the average width of the running band. We measured the inner radii *r*_1_ and the widths *d* of a running band of each stone with a calliper at four places at 90° intervals. The value *r*_1_ ranged from 122 (A1, A2) to 126 mm (D1, D2), and the value *d* ranged from 3.05 ± 0.02 (D1) to 6.20 ± 0.00 mm (A2).

Figure 5b shows the relationship between the *SRA* defined by Eq. (8) and the curl distance for an ordinary pebbled ice surface shown by solid circles and a solid line, and for a flat ice surface shown by open circles and a broken line. The regression lines are given by

9 $${x}_{\mathrm{p}}= 0.0092 SRA+0.116$$

10 $${x}_{\mathrm{f}}= 0.0093 SRA+0.248.$$

We find that the relationship is better than the relationship in Fig. 5a; the correlation coefficients (*r*) and the level of significance (*p*) are 0.97 (*p* < 0.01) and 0.98 (*p* < 0.01). Because the *SRA* is corrected for the different width of the running band for each stone, the relationship between the curl distance and the surface roughness condition of running bands is better as shown in Fig. 5b.

We also find that the different ice surface condition contributes to the curl distances of 0.13 to 0.15 m for different values of *SRA* from 10 to 150 μm cm^2^. Thus, the ice surface condition has much smaller effects on the curl distance than the surface roughness condition of curling stones, because the flat ice surface condition in Fig. 1d is an unusual surface condition, and similar pebbled ice surfaces as shown in Fig. 1c are usually used in ordinary curling games.

As already mentioned, the curling distances of curling stones are different on every curling rink. The reason has been unknown to curling players and to scientists studying the curling phenomena of a stone sliding on ice. Most curling players may consider that the difference is primarily caused by ice surface conditions, including ice surface temperature, the number density of pebbles, and the diameter and height conditions of pebbles. However, our study clearly demonstrates that the curl distance is primarily determined by the surface roughness and the surface area of the running band on the bottom of a stone, and the ice surface condition has smaller effects on the curl distance. We expect that our findings will form the basis of future curling theories and model calculations regarding the curling phenomenon of curling stones and will clarify requirements of the running band on the bottom of a stone to achieve an appropriate curl distance in curling games.

Using the results in this study, ice technicians in curling halls can adjust the curl distance to 1.0 m or 1.5 m with an ordinary angular velocity (4 turns from delivery to stop) by setting the average surface roughness (*R*_a_) of a running band to 2.32 μm for a curling distance of 1.0 m and to 3.54 µm for a curling distance of 1.5 m, according to Eq. (6). Because the *SRA* is 41.4 μm cm^2^ or 42.5 μm cm^2^ for these curl distances, the width of the running band is to be 5.2 mm or 5.3 mm if the inner diameter of the running band is 124 mm, according to Eq. (9). Thus, the curl distance of a stone sliding on ice in every curling hall can be adjusted for an appropriate value by changing the surface roughness of the running band on the bottom of a stone. The surface ice temperature and pebbled ice surface conditions in curling rinks should be the same as described in this paper.

Finally, we propose a possible frictional mechanism affecting the curling phenomena of a curling stone based on our results. Our results reveal that the amount of curl, or specifically the lateral displacements of a stone, increase with the increase of the surface roughness and the area of the running band. We consider that the increase of the surface roughness and the area causes the increase of erosion depths of pebbles, which leads to larger ploughing forces acting at the contact points between the running band and the surface of ice pebbles. Thus, we consider that inhomogeneous distribution of the ploughing forces around the running band or continuous small pivoting around the contact points possibly causes the curling phenomena of a curling stone moving on ice. These are the reasons for the FB model and the PS model, respectively.

## Conclusion

In this paper, we clarified the following relating to the curl distance of a stone moving on ice.

1. The curl distance of a stone is primarily determined by the surface roughness of a running band of a stone. Using the products of the surface roughness and the area of the running band (*SRA* in this paper), the relationship was improved. This shows the importance of the surface roughness and the area of the running band for the curling phenomenon of a stone moving on ice.
2. With the increase of the surface roughness of the running band, erosion depths at the surface of pebbles will be deeper, and larger ploughing forces are generated at the contact points between the running band and the surface of pebbles. Inhomogeneous distribution of the ploughing forces around the running band or continuous small pivoting around the contact points will possibly cause the curling phenomena of a curling stone moving on ice. The former is the reason for the FB model, and the latter is the reason for the PS model.
3. The curl distance of a stone sliding on ice in every curling hall can be adjusted to an appropriate value by changing the surface roughness of the running band on the bottom of a stone, under the same conditions as described in this paper for the surface ice temperature and the pebbled ice surface conditions in curling rinks.
4. The ice surface conditions contribute to the curling distance of a stone. However, the effect was smaller than that of the surface roughness and the *SRA* of a stone.

## Data Availability

The datasets generated from this study are available from the corresponding author on reasonable request.
